# The Feasibility of Ultrasonography in Defining the Size of Jaw Osseous Lesions

**Published:** 2015-12

**Authors:** Shoaleh Shahidi, Alireza Shakibafard, Barbod Zamiri, Mohammad Reza Mokhtare, Maneli Houshyar, Soroush Mahdian

**Affiliations:** a Biomaterial Research Center, Dept. of Oral Radiology, School of Dentistry, Shiraz University of Medical Sciences, Shiraz, Iran.; b TABA Medical Imaging Center, Shiraz, Iran.; c Dept. of Oral Surgery, School of Dentistry, Shiraz University of Medical Sciences, Shiraz, Iran.; d Dept. of Oral Radiology, School of Dentistry, Shiraz University of Medical Sciences, Shiraz, Iran.; e Dept. of Oral Radiology, School of Dentistry, Arak University of Medical Sciences, Arak, Iran.; f Undergraduate Student, School of Dentistry, Arak University of Medical Sciences, Arak, Iran.

**Keywords:** Ultrasonography, Tomography, X-ray Computed, Odontogenic Tumors, Odontogenic Cyst, Jaw

## Abstract

**Statement of the Problem:**

Jaw bone lesions are common pathologic conditions. The role of ultrasonography in evaluation of the extra-osseous lesions is confirmed, however, this imaging modality is not the diagnostic routine for the intra-osseous jaw lesions.

**Purpose:**

The purpose of this study was to evaluate the efficiency of ultrasonography in diagnosis of intra-osseous jaw lesions concerning their size and content and also to study its correlation with the histopathological findings.

**Materials and Method:**

For this study, 15 patients with intra-osseous jaw lesions in the maxilla and mandible were selected from those referred to the Department of Oral Surgery. Panoramic imaging, computed tomography (CT) or cone beam computed tomography (CBCT) and ultrasonography (USG) were performed for all the lesions. The size of the lesions was measured by USG and then compared with CT or CBCT. Moreover, the correlation amongst the echographic patterns and histopathologic results was evaluated.

**Results:**

In 12 cases, size values were in complete agreement with CT or CBCT. The size of 3 lesions could not be measured by the radiologist due to the thickness of buccal cortical plate.

**Conclusion:**

Findings of this study suggested that USG might be feasible in estimating the size of intra-osseous jaw lesions with little underestimation. This study also confirmed that ultrasound imaging was a very useful imaging technique which could provide significant diagnostic information regarding the content of jaw bone lesions where the buccal bone thickness was thin enough.

## Introduction

Imaging techniques play a crucial role in detecting, diagnosis, treatment and follow-up assessments of intra-osseous maxillofacial lesions. Because of the wide variation of jaw bone lesions, the diagnosis is often complex.[1-2]

Following the advancements in technology, various imaging modalities have been introduced to professional use in this field; however, panoramic radiography is still the gold standard for the first-step two-dimensional assessment of jaw lesions among other conventional techniques.[3] Despite its high radiation dose, Computed Tomography (CT) scan is being used regularly as an advanced complementary method in diagnosis of jaw bone lesions.[4]

Cone beam computed tomography (CBCT) is a more recent alternative to panoramic radiography with the benefit of lower radiation exposure.[5]Meanwhile, ultrasonography (USG) has been frequently used in evaluating the solid and cystic nature of the lesions. Although this technology is safe and non-invasive, its use in dental practice has been limited to soft tissues.[4]

The number of studies investigating the role of ultrasound in evaluation of bony lesions is limited. In 1996, Lauria *et al.*[6] prospectively evaluated the role of ultrasonography as a complementary imaging modality in the diagnosis of intra-osseous jaw lesions. They concluded that USG was a useful technique in evaluation of the content of lesions. Cotti *et al.* in 2002, 2003[1-2] and Gundappa *et al.* in 2006[7] assessed whether combined use of USG and color Doppler could differentiate the periapical lesions based on their content. They concluded that USG was a useful technique to distinguish between cyst and granuloma by revealing the content of the bony lesion. Sumer *et al.* in 2009,[4] suggested that USG provided accurate information on the content of intra-osseous jaw lesions and Doppler ultrasound was capable of showing vascularization of such lesions.

The purpose of our study was to evaluate the intra-osseous jaw bone lesions by means of conventional radiography, CT or CBCT scans, and USG regarding the size and content of these lesions and to compare the correlation between the ultrasonographic findings and histopathologic results. 

## Materials and Method

Out of the patients referring to maxillofacial surgeons in Shiraz, 15 cases with intraosseous jaw lesions in the maxilla or mandible were enrolled in this study. After receiving approval from the 

Ethics Committee, all the patients consented to participate in the study and were informed about the techniques used and any potential risks or benefits. 

An expert dental radiologist and an expert radiologist-each with a minimum of 15 years of clinical experience- were recruited as observers and to take measurements from the images obtained. After the clinical examination and taking panoramic radiographs (Planmeca2010; ProlineXC, Helsinki, Finland) that confirmed the presence of lesion in maxilla or mandible, CBCT (Kodak 9000; Carestream, Rochester, NY, USA) with field of view of 5×4 cm, CBCT Newtom VGi (QR srl; Verona, Italy) with minimum and maximum field of view of 6×6 up to 15×15, or spiral CT scan (GE; Milwaukee, United States of America) was taken. The radiographs were viewed and evaluated on a viewing box under normal operating illumination. Panoramic images with low quality conditions were excluded from the project.

On order to detect lesions and to evaluate their size and content, ultrasound examination was performed for all patients by means of Medison ultrasound unit (Samsung; Medicon Co.Ltd., Seoul, South Korea) with a 5-13 MHz linear-array probe of 40mm or convex-array probe of 80mm (for lesions>5cm). The ultrasound probe was positioned outside the mouth on the skin overlying the intra-osseous lesion while USG evaluation was performed real-time, measuring the specific slice in which the lesion appeared with its largest dimensions. The USG survey was performed in a room with flexible light on a high-resolution 19-inch monitor of Medison unit. The radiologist was blinded to the previous radiographs. All the patients underwent a biopsy followed by surgical treatment and the specimens were submitted for histopathologic evaluation where a definitive diagnosis was made.  **

Size of all lesions was initially analyzed by using CT or CBCT. Measurements in CBCT scans were made using accurate inherent software by a maxillofacial radiologist with a precision of 0.1mm.

The size of the lesions in CT scans was measured by the scale supported for all slices. Any measurement was applied on the specific slice in which the lesion appeared with its largest dimensions. 

An expert radiologist also measured the lesion size in the specific USG slice in which the lesion manifested its greatest dimension. The aforementioned data was then compared with CT or CBCT as gold standards. 

Intraclass correlation coefficient (ICC) between both modalities (US and CT) was then evaluated to assess their agreement regarding size evaluation.

To facilitate the comparison with histopathologic results, USG findings were classified into the following 5 groups; anechoic: characteristic of cystic lesions because of liquid content; hypoechoic: characteristic of cystic lesions with dense liquid content (e.g., odontogenic keratocyst) and odontogenic tumors with semisolid content; hyperechoic: characteristic of solid tumors with solid content; mixed echogenic: characteristic of solid odontogenic lesions consisting both cystic and solid components, irregular hyperechoic pattern inside anechoic area, and a unique sonographic appearance of lesions consisting air inside.

According to these echographic patterns, jaw lesions in this study were categorized into three groups: cystic (liquid content) manifesting anechoic pattern with posterior enhancement, semisolid (dense liquid content) showing mixed echoic pattern or hypoechoic pattern, and solid illustrating hyperechoic pattern without any posterior enhancement. Echographic pattern of all the lesions was then compared with histopathologic results. 

## Results

In this study, 15 intraosseous jaw lesions were assessed in 15 patients. Ten lesions were located in the mandible and 5 in the maxilla. The size measurement data of all lesions analyzed by CBCT/CT is displayed in Table 1.

**Table 1:** CBCT / CT scan and USG measurement of jaw lesions

**Type of Lesion**	**CBC T or CT (Cm)**	**USG (Cm)**	**Location**
Residual cyst	1.61 x 1.12	1.18 x 0.98	Post maxilla
Radicular cyst	1.83 x 1.03	1.42 x 0.62	Post maxilla
Radicular cyst	2.44 x 0.98	2.03 x 0.64	Ant maxilla
Radicular cyst	1.91 x 0.88	1.88 x 0.81	Body mandible
Infected radicular cyst	3.0 x 1.08	2.21 x 0.91	Ant maxilla
Infected residual cyst	5.40 x 4.50	5.36 x 4.14	Ant mandible (canine)
Dentigerous cyst	3.05 x 2.00	2.82 x 1.93	Body mandible
Odontogenic kerato cyst	3.21 x 2.22	2.81 x 1.74	Ant maxilla
Simple bone cyst	Inconclusive	Inconclusive	Body mandible
Mural ameloblastoma	5.55 x 3.00	5.56 x 2.48	Body mandible
Ameloblastoma	5.88 x 4.37	5.84 x 4.04	Ramus mandible
Odontogenic myxoma	4.43 x 2.67	Inconclusive	Body mandible
Pindborg tumor(CEOT)	3.20 x 1.50	2.88 x 1.47	Body mandible
Central giant cell granuloma	2.94 x 2.730	2.57 x 2.32	Premolar mandible
Central giant cell granuloma	5.55 x 3.77	Inconclusive	Molar mandible

As stated in Table 2, ICC between the two modalities (US and CT) was 0.99; indicating complete agreement between them. The radiologist could not measure the whole lesion size in three of the cases due to the thickness of buccal cortical plate. Distribution of the lesions by histopathologic results regarding their echographic pattern is demonstrated in Table 3.

**Table 2:** Assessment of agreement between the two modalities (CT and USG) in measurement of length and width

**Variable**	**Intraclass Correlation** **Coefficient**	**95%Confidence** **Interval**
Length	0.99	(0.96-0.99)
Width	0.99	(0.97-0.99)

**Table 3:** Histopathologic type, radiologic and echographic pattern of each lesion

**Histopathological Type**	**Panoramic**	**CT or CBCT**	**USG**
Residual cyst	Radiolucent	Radiolucent	Anechoic
Radicular cyst	Radiolucent	Radiolucent	Anechoic
Radicular cyst	Radiolucent	Radiolucent	Anechoic
Radicular cyst	Radiolucent	Radiolucent	Anechoic
Infected radicular cyst	Radiolucent	Radiolucent	Hypoechoic
Infected residual cyst	Radiopaque	Radiolucent	Hypoechoic
Dentigerous cyst	Radiolucent	Radiolucent	Anechoic
Odontogenic kerato cyst	Radiolucent	Radiolucent	Hypoechoic
Simple bone cyst	Radiolucent	Radiolucent	Irregular hyperechoic
Mural ameloblastoma	Radiolucent	Radiolucent	Hyperechoic
Ameloblastoma	Mixed radiopaque radiolucent	Mixed radiopaque radiolucent	Hyperechoic
Odontogenic myxoma	Mixed radiopaque radiolucent	Mixed radiopaque radiolucent	Mixed echoic
Pindborg tumor (CEOT)	Radiolucent	Mixed radiopaque radiolucent	Hyperechoic
Central giant cell granuloma	Mixed radiolucent radiopaque	Radiolucent	Mixed echoic
Central giant cell granuloma	Radiolucent	Mixed radiopaque radiolucent	Mixed echoic

## Discussion

As stated previously, only a limited number of studies have investigated the role of ultrasound in diagnosis of jaw bone lesions, particularly in evaluation of the size of these lesions. Our findings regarding the content of lesions were comparable with Lauria *et al.*[6] and Sumer *et al.*[4] who concluded that USG could provide accurate information on the content of intraosseous jaw lesions before any surgical procedure. Neither of these studies provided information on the size of lesions comparing USG with CT or CBCT.

To the best of our knowledge, this study is the first one that has simultaneously evaluated the size and content of the jaw lesions by means of USG and compared it with gold standards (CT or CBCT) and histopathological results. The size of lesions was more regarded in this study.

Since CT and CBCT are the gold standards in lesion size assessment,[8] the measurements obtained from USG were compared with those of CT and CBCT. In 9 out of 15 cases in which the lesions were measured by linear probe of USG with the frequency of 7.5 MHz, sizes had negligible differences in comparison with CT /CBCT results.

In 3 other cases in which the size of lesions was larger than 5 cm, convex probe was used to cover a larger field of view that could measure the whole lesion. In these cases the results were very close to CT or CBCT results.

In three cases, because of thickness of buccal cortical plate, the radiologist could not measure the lesions accurately. The loss of continuity of buccal cortex, not the content of these lesions, was more important in assessment of the size of lesions in USG. 

All measurements were underestimated using USG compared with CT or CBCT. Based on our findings, this might be due to the projection of acoustic shadow of the bony edges on the lateral walls. Therefore, placement of the electronic calipers for exact measurements is difficult. As mentioned previously, ICC between the two modalities (US and CT) was 0.99, confirming their complete agreement.

**Figure 1:**
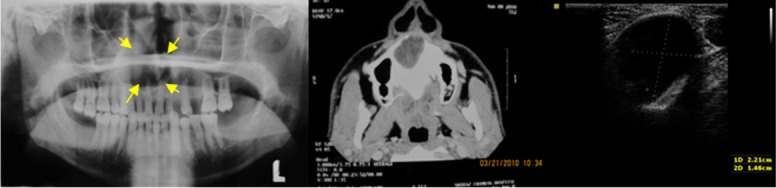
A case of radicular cyst. (A) Panoramic view reveals a radiolucent lesion on the anterior right side of the maxilla. (B) CT scan (tissue level) shows lytic lesion in the maxilla. (C) Ultrasound image shows anechoic lesion without internal echoes.

Echographic pattern in all these lesions were evaluated with USG. Five out of 15 lesions showed anechoic pattern (no internal echoes) with respect to their cystic content. These odontogenic cysts included radicular, residual, and dentigerous cysts (Figure 1).

**Figure 2:**
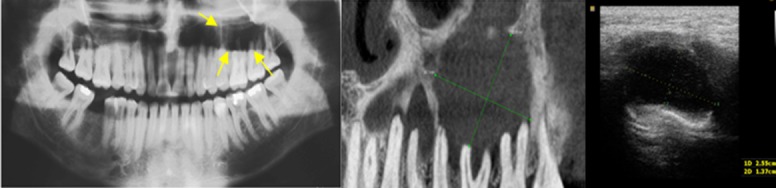
A case of odontogenic keratocyst. (A) Panoramic view reveals a radiolucent lesion in left posterior region of the maxilla. (B) CBCT shows lytic lesion and a prominent septum inside the lesion. (C) Ultrasound image shows a well demarcated hypoechoic lesion.

A hypoechoic area is an area on the image with fewer reflected echoes, darker than the surrounding tissues which can be an indication of a semi-solid content.[9-11] Three lesions revealed a hypoechoic image based on the nature of their content which was infected radicular cysts and odontogenic keratocyst (OKC). Histologically, these two types of cysts contain a denser liquid compared to other cysts. Higher viscosity might be due to pus accumulation in the infected radicular cyst and the keratin content in OKC. Therefore, their hypoechogenicity in comparison with other cysts (anechogenicity) may confirm this internal viscosity (Figure 2). Based on the oral radiology textbooks, the density or viscosity of keratin contents of OKC would not affect the internal architecture of this lesion in conventional radiographs; however, USG had the advantage of differentiating these lesions with respect to these changes in keratin viscosity.

**Figure 3:**
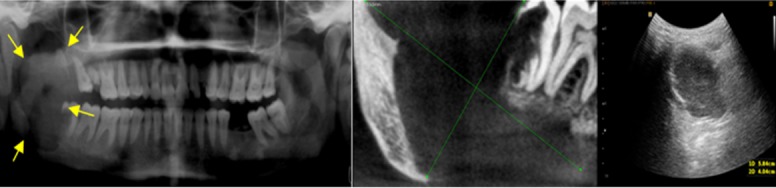
A case of ameloblastoma. (A) Panoramic view reveals large radiolucent lesion in right mandibular ramus. (B) CBCT shows large lytic lesion in right ramus. (C) Ultrasound image shows a hyperechoic lesion.

A hyperechoic area has higher echo intensity (demonstrates more reflected echoes) than the surrounding tissues, and it almost always indicates solid content. Three lesions which revealed a hyperechoic pattern were ameloblastoma, mural ameloblastoma, and Pindborg tumor (calcifying epithelial odontogenic tumor). These tumors had a solid content which was consistent with their histopathologic results (Figure 3).

**Figure 4:**
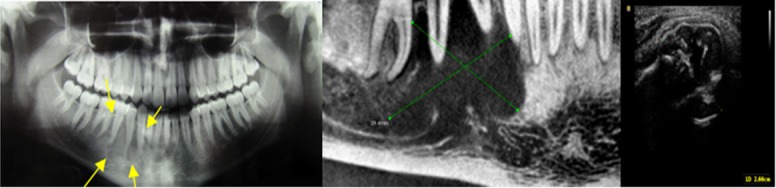
A case of central giant cell granuloma. (A) Panoramic view reveals radiolucent lesion with scalloped margin in the right mandibular body. (B) CBCT shows lytic lesion in right mandibular body. (C) Ultrasound image shows mixed echogenicity (hyperechoic-hypoechoic pattern)

USG pattern of three lesions including two central giant cell granulomas and one odontogenic myxoma was hyperechoic-hypoechoic (mixed echogenic), indicating solid and mixed content of these lesions[9, 12](Figure 4).

**Figure 5:**
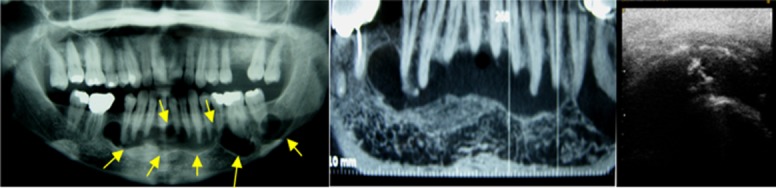
A case of simple bone cyst. (A) Panoramic view reveals extensive radiolucent lesion with scalloped border in left mandible. (B) CBCT shows large lytic lesion in left mandible. (C) Ultrasound image shows irregular hyperechoic pattern presenting air inside the lesion.

Based on our findings, the irregular hyperechoic pattern in anechoic area detected in the case of simple bone cyst was highly suggestive of air similar to the echographic appearance of the air in other parts of the body such as lungs.[10, 13-14] Based on review of the literature, there has been no report of this unique appearance in the USG findings of jaw lesions prior to this study (Figure 5).

In all the aforementioned cases, there was complete agreement between the USG and histopathologic results regarding content assessment.

Ultrasound does not eliminate the necessity of using invasive procedures namely aspiration, incisional and excisional biopsies to obtain final diagnosis for intraosseous lesions. However, based on our findings this safe modality is highly recommended whenever possible (i.e., the thin buccal cortical plate permits the penetration of sound waves) prior to any invasive diagnostic procedure for evaluation of intraosseous jaw lesions in size and content. USG is not routinely used to evaluate the size or content of the intraosseous jaw lesions in the literature, therefore, the number of cases in this study seems to be reasonable. Future studies with bigger sample size would help disclosing more feasible aspects of USG in assessment of jaw osseous lesions. 

## Conclusion

Findings of this study suggest that USG might be feasible in estimating the size of intraosseous jaw lesions with an ignorable underestimation. This study confirms that ultrasound imaging is a very useful imaging technique which can give significant diagnostic information regarding the content of jaw bone lesions where the buccal bone thickness is thin enough. Further studies in this field seem inevitable to clarify ultrasound findings. 
